# Decoction of Oats as a Diuretic

**Published:** 1855-03

**Authors:** 


Decoction of Oats as a Diuretic.—Many years ago, Dr. Themont called the attention of medical men to the remarkable diuretic properties of decoction of oats. Although the paper announcing his observation contained the narrative of a case of cardiac dropsy cured by the sole use of this remedy, yet he did not succeed in exciting much interest on the part of the profession in his discovery. We have seen the oat-tea tried pretty frequently of late in cases of dropsy, in most of them in combination with other treatment, but unassisted in a sufficient number to fairly test its virtues. Its powers are probably not at all superior to those of the decoction of broom; and as a good alternating remedy with the latter, its proper place in therapeutics would perhaps be assigned. Its simplicity and freedom from injurious qualities are great recommendations, since it may, without risk, be entrusted as a domestic remedy to patients not under regular care. In several cases of slight œdema of the extremities, consequent on heart disease, the patients succeeded, by its use alone, in getting rid of that symptom. The mode of preparation is to take two handfuls of common oats (not in any way prepared,) and boil them in three quarts of water for about a quarter of an hour. Of the strained decoction, a teacupful should be given frequently, as an ordinary drink.—*Med. Times and Gaz.* Sept. 9, 1854.

